# Supplementary material for: Construction of a prognostic prediction model for concurrent radiotherapy in cervical cancer using GEO and TCGA databases with preliminary validation analysis
Source: PLoS One. 2025 Oct 31;20(10):e0334281. doi: 10.1371/journal.pone.0334281 (PMC12578209; doi:10.1371/journal.pone.0334281)
Supplement: S1 Data — Gene expression profiles and corresponding clinical data of TCGA patients were obtained from https://portal.gdc.cancer.gov/. Original dataset GSE236738. The original data supporting the findings of this study can be accessed at https://www.ncbi.nlm.nih.gov/geo/query/acc.cgi?acc=GSE236738. Additional GEO dataset. Additional raw data are available at https://www.ncbi.nlm.nih.gov/geo/query/acc.cgi. (ZIP) [file pone.0334281.s001.zip › support/source code.docx]

install.packages("Seurat")

#scRNA-seq

rm(list = ls());gc()

library(Seurat)

setwd(set)

dir <- list.files()

dir

num <- data.frame(matrix(ncol = 3, nrow = 6))

colnames(num) <- c("Sample","features","cells")

num$Sample <- dir

for(i in 1:6){

setwd <- paste0(set,"\\",dir[i])

data <- Read10X(data.dir = setwd)

seurat <- CreateSeuratObject(counts = data, project = dir[i],

min.cells = 3, min.features = 200)

rm("data");gc()

saveRDS(seurat,file = paste0(dir[i],".RDS"))

data <- as.data.frame(seurat[["RNA"]]$counts)

num1 <- length(colnames(data))

num2 <- length(rownames(data))

num$cells[i] <- num1

num$features[i] <- num2

rm("data");gc()

rm("seurat");gc()

}

write.table(num,"num.csv",sep=",",row.names=F)

rm(list = ls());gc()

library(DoubletFinder)

library(tidyverse)

library(patchwork)

library(Seurat)

dir <- list.files()

dir

for (i in 1:6) {

seu_kidney <- readRDS(dir[i])

seu_kidney <- NormalizeData(seu_kidney)

seu_kidney <- ScaleData(seu_kidney)

seu_kidney <- FindVariableFeatures(seu_kidney, selection.method = "vst", nfeatures = 2000)

seu_kidney <- RunPCA(seu_kidney)

seu_kidney <- RunUMAP(seu_kidney, dims = 1:10)

sweep.res.list_kidney <- paramSweep(seu_kidney, PCs = 1:10, sct = FALSE)

sweep.stats_kidney <- summarizeSweep(sweep.res.list_kidney, GT = FALSE)

bcmvn_kidney <- find.pK(sweep.stats_kidney)

annotations <- seu_kidney@meta.data$ClusteringResults

homotypic.prop <- modelHomotypic(annotations)

number <- 0.075*length(seu_kidney@meta.data$orig.ident)

nExp_poi <- round(number)

nExp_poi.adj <- round(nExp_poi*(1-homotypic.prop))

seu_kidney <- doubletFinder(seu_kidney, PCs = 1:10, pN = 0.25,

pK = 0.09, nExp = nExp_poi.adj,

reuse.pANN = F, sct = T)

seu_kidney@meta.data$double <- seu_kidney@meta.data[[5]]

seu_kidney@meta.data[[5]] <- NULL

seu_kidney@meta.data[[4]] <- NULL

seu_kidney <- subset(seu_kidney,double == "Singlet")

seu_kidney$double <- NULL

saveRDS(seu_kidney,

file = paste0("E:\\desktop\\else\\CC\\scRNA-seq\\remove double quotes\\",

dir[i]))

}

rm(list = ls());gc()

library(Seurat)

setwd("E:\\desktop\\else\\CC\\scRNA-seq\\remove double quotes\\")

dir <- list.files()

dir

num <- data.frame(matrix(ncol = 3, nrow = 6))

colnames(num) <- c("Sample","features","cells")

num$Sample <- dir

for (i in 1:length(dir)) {

pbmc <- readRDS(dir[i])

pbmc[["percent.mt"]] <- PercentageFeatureSet(pbmc, pattern = "^MT")

pbmc <- subset(pbmc,

subset = nFeature_RNA > 200 & nFeature_RNA < 5000 &

percent.mt < 10)

data <- as.data.frame(pbmc[["RNA"]]$counts)

num1 <- length(colnames(data))

num2 <- length(rownames(data))

num$cells[i] <- num1

num$features[i] <- num2

saveRDS(pbmc,

paste0(file = "E:\\desktop\\else\\CC\\scRNA-seq\\quality control\\",

dir[i]))

rm(pbmc);gc()

}

write.table(num, "E:\\desktop\\else\\CC\\scRNA-seq\\quality control\\num.csv",

sep=",",row.names=F)

rm(list = ls());gc()

library(Seurat)

#健康组

setwd "E:\\desktop\\else\\CC\\scRNA-seq\\quality control\\before")

dir <- list.files()

dir

names <- c("GSM7574777","GSM7574779","GSM7574781")

scRNAlist <- list()

for(i in 1:length(dir)){

scRNAlist[[i]] <- readRDS(dir[i])

scRNAlist[[i]]@meta.data$Sample <- names[i]

scRNAlist[[i]]@meta.data$orig.ident <- "TN"

}

names(scRNAlist) <- names

scRNA <- merge(x=scRNAlist[[1]], y=scRNAlist[2:length(scRNAlist)])

dim(scRNA)

table(scRNA@meta.data$orig.ident)

table(scRNA@meta.data$Sample)

saveRDS(scRNA,file="TN.RDS")

setwd("E:\\desktop\\else\\CC\\scRNA-seq\\quality control\\after")

dir <- list.files()

dir

names <- c("GSM7574778","GSM7574780","GSM7574782")

scRNAlist <- list()

for(i in 1:length(dir)){

scRNAlist[[i]] <- readRDS(dir[i])

scRNAlist[[i]]@meta.data$Sample <- names[i]

scRNAlist[[i]]@meta.data$orig.ident <- "CCRT"

}

names(scRNAlist) <- names

scRNA <- merge(x=scRNAlist[[1]], y=scRNAlist[2:length(scRNAlist)])

dim(scRNA)

table(scRNA@meta.data$orig.ident)

table(scRNA@meta.data$Sample)

saveRDS(scRNA,file="CCRT.RDS")

#

rm(list = ls());gc()

setwd("E:\\desktop\\else\\CC\\scRNA-seq\\quality control")

TN <- readRDS("TN.RDS")

CCRT <- readRDS("CCRT.RDS")

merge <- merge(TN,CCRT)

saveRDS(merge,"merge.RDS")

install.packages("harmony")

rm(list = ls());gc()

library(harmony)

library(Seurat)

library(tidyverse)

setwd("E:\\desktop\\else\\CC\\scRNA-seq\\quality control")

pbmc <- readRDS("merge.RDS")

pbmc <- NormalizeData(pbmc) %>% FindVariableFeatures() %>% ScaleData() %>% RunPCA(verbose = FALSE)

pbmc <- RunHarmony(pbmc, group.by.vars = "Sample")

ElbowPlot(pbmc, ndims = 30, reduction = "pca")

pbmc <- RunUMAP(pbmc, reduction = "harmony", dims = 1:10)

pbmc <- FindNeighbors(pbmc, reduction = "harmony", dims = 1:10) %>% FindClusters(resolution = 0.3)

DimPlot(pbmc, reduction = "umap", group.by = "orig.ident")

DimPlot(pbmc, reduction = "umap", group.by = "Sample")

DimPlot(pbmc, reduction = "umap", label = TRUE, repel = TRUE)

saveRDS(pbmc,"harmony03.RDS")

rm(list = ls());gc()

library(Seurat)

setwd("E:\\desktop\\else\\CC\\scRNA-seq\\quality control")

pbmc <- readRDS("harmony03.RDS")

VlnPlot(pbmc, features = "nFeature_RNA",group.by = "Sample",pt.size = 0)+NoLegend()

VlnPlot(pbmc, features = "nCount_RNA",group.by = "Sample",y.max = 40000,pt.size = 0)+NoLegend()

VlnPlot(pbmc, features = "percent.mt",group.by = "Sample",pt.size = 0)+NoLegend()

VlnPlot(pbmc, features = c("CD2","CD3E","GZMA","TRAC","CD3D"))

VlnPlot(pbmc, features = c("KRT5","KRT13","KRT16"))

VlnPlot(pbmc, features = c("AIF1","CD68","CD163","MARCO","MSR1","CD14"))

VlnPlot(pbmc, features = c("CD79A","CD79B","MS4A1","CD19","CR2"))

VlnPlot(pbmc, features = c("COL3A1","LUM","DCN"))

VlnPlot(pbmc, features = c("TPSB2", "TPSAB1","CPA3"))

VlnPlot(pbmc, features = c("CD34", "VWF","CLDN5","PECAM1"))

new.cluster.ids <- c("T cells", "T cells", "T cells", "Tumor cells", "Macrophages",

"Macrophages", "B cells", "Tumor cells", "T cells",

"Fibroblast", "Tumor cells", "Mast cells", "B cells",

"Fibroblast", "T cells", "Endothelial cells", "Tumor cells")

names(new.cluster.ids) <- levels(pbmc)

pbmc <- RenameIdents(pbmc, new.cluster.ids)

DimPlot(pbmc, reduction = "umap", label = TRUE, pt.size = 1) +NoLegend()

pbmc$clusters <- Idents(pbmc)

saveRDS(pbmc, file = "rename.RDS")

table(Idents(pbmc))

prop.table(table(Idents(pbmc)))

Cellratio <- prop.table(table(Idents(pbmc), pbmc$Sample), margin = 2)

Cellratio <- as.data.frame(Cellratio)

write.table(Cellratio, "Cellratio.csv", sep = ",", row.names = F)

markers.to.plot <- c("CD2","CD3E","TRAC","CD3D",

"KRT18","CLDN4","KRT8","EPCAM",

"AIF1","CD68","CD14","CD163","MSR1",

"CD79A","CD79B","MS4A1","CD19","CR2",

"COL3A1","LUM","DCN",

"TPSB2", "TPSAB1","CPA3",

"PECAM1","VWF","CD34","CLDN5")

DotPlot(pbmc, features = markers.to.plot) +

RotatedAxis()

Tumor <- subset(pbmc,clusters == "Tumor cells")

Tumor

saveRDS(Tumor, file = "Tumor.RDS")

rm(list = ls());gc()

library(Seurat)

setwd("E:\\desktop\\ else\\CC\\scRNA-seq\\quality control")

Tumor <- readRDS("Tumor.RDS")

table(Tumor$orig.ident)

Idents(Tumor) <- Tumor$orig.ident

Tumor$clusters <- Tumor$orig.ident

Tumor <- JoinLayers(Tumor)

markers <- FindAllMarkers(Tumor, only.pos = TRUE)

write.table(markers,"markers.csv",sep = ",",row.names = F)

markers %>%

group_by(cluster) %>%

slice_head(n = 10) %>%

ungroup() -> top10

DoHeatmap(Tumor, features = top10$gene) + NoLegend()

Tumor <- ScaleData(object = Tumor, features = rownames(Tumor))

subobj <- subset(Tumor, downsample = 1000)

DoHeatmap(subobj, features = top10$gene)+ NoLegend()

setwd("E:\\desktop\\else\\CC\\data")

library(AnnoProbe)

idprob = AnnoProbe::idmap("GPL8490",type = 'soft')

GPL8490 <- getGEO("GPL8490", destdir=".")

GPL8490 <- GPL8490[[1]]

library(GEOquery)

gset = getGEO('GSE30759', destdir=".", AnnotGPL = F, getGPL = F)

exp<-exprs(gset[[1]])

exp <- as.data.frame(exp)

expr <- filterEM(exp,idprob )

write.table(exp,"GSE30759.csv",sep = ",",row.names= T)

pdata <- pData(gset[[1]])

write.table(pdata,"GSE30759_clinic.csv",sep = ",",row.names= T)

CESC <- read.table("GSE30759.csv",sep = ",",header = T,check.names = F)

gene <- read.table("gene.csv",sep = ",",header = T,check.names = F)

GEO <- merge(gene,CESC,by = "ID")

write.table(GEO,"GSE30759.csv",sep = ",",row.names= F)

rm(list = ls());gc()

CESC <- read.table("GSE56363.csv",sep = ",",header = T,row.names = 1,check.names = F)

View(CESC)

library(limma)

library(dplyr)

library(tidyverse)

list <- c(rep("NCR", 9),rep("CR",12)) %>%

factor(., levels = c("NCR", "CR"), ordered = F)

list

list <- model.matrix(~factor(list)+0)

list

colnames(list) <- c("NCR", "CR")

list

df.fit <- lmFit(CESC, list)

df.fit

df.matrix <- makeContrasts(NCR - CR, levels = list)

df.matrix

fit <- contrasts.fit(df.fit, df.matrix)

fit <- eBayes(fit)

tempOutput <- topTable(fit,n = Inf, adjust = "BH")

tempOutput$gene <- rownames(tempOutput)

write.table(tempOutput,"NCRvsCR.csv",

sep = ",",row.names= F)

rm(list = ls());gc()

#

library(stringr)

library(enrichplot)#GO,KEGG,GSEA

library(clusterProfiler)#GO,KEGG,GSEA

GO_database <- 'org.Hs.eg.db'

#

setwd("D:\\Desktop Files\\TCGA\\DEGs")

dir <- list.files()

dir

LumA <- read.table(file.choose(),sep = ",",header = F)

gene <- LumA[,1]

gene <- bitr(gene,

fromType = 'SYMBOL',

toType = 'ENTREZID',

OrgDb = GO_database)

GO <-enrichGO(gene$ENTREZID,

OrgDb = GO_database,

keyType = "ENTREZID",

ont = "BP"

pvalueCutoff = 0.05,#设定p值阈值

qvalueCutoff = 0.05,#设定q值阈值

readable = T)

result <- GO@result

result <- as.data.frame(result)

write.table(result,"all.csv",sep = ",",row.names = F)

HER2 <- read.table(file.choose(),sep = ",",header = T)

HER2$pvalue <- -log10(HER2$pvalue)

HER2$pvalue <- as.numeric(HER2$pvalue)

ggplot(HER2,

aes(x = Count,

y = reorder(Description,pvalue),

fill = pvalue))+

geom_bar(stat = "identity")+

theme_bw()+

scale_fill_gradient(low="blue",high ="red")+

labs(y = NULL)+

theme(axis.text = element_text(face="bold",size = 12))+

theme(

plot.margin = margin(t = 20

r = 20

b = 20

l = 20))

install.packages("ggvenn")

library(ggvenn)

a <- read.table(file.choose(),sep = ",",header = T,check.names = F)

b <- list("GSE56363(NCRvsCR)" = a$`GSE56363(NCRvsCR)`,

"GSE236738(CCRTvsTN)" = a$`GSE236738(CCRTvsTN)`)

ggvenn(b, c("GSE236738(CCRTvsTN)", "GSE56363(NCRvsCR)"),

show_percentage = F,

stroke_color = "white",

fill_color = c("#E41A1C","#1E90FF","#FF8C00",

"#4DAF4A","#984EA3"),

set_name_color =c("#E41A1C","#1E90FF"))

setwd("E:\\desktop\\else\\CC\\data")

dir <- list.files()

library(data.table)

data <- fread("TCGA-GTEx-TARGET-gene-exp-counts.deseq2-normalized.csv",

sep = "\t",header = T)

sample <- read.table("sample.csv",sep = ",",header = F,check.names = F)

sample <- sample[,1]

expression <- dplyr::select(data,sample)

write.table(expression,"CESC-GTEX-154.csv",sep = ",",row.names= F)

rm(list = ls());gc()

setwd("E:\\desktop\\else\\CC\\data")

CESC <- read.table("CESC-GTEX-154.csv",sep = ",",header = T,row.names = 1,check.names = F)

library(tinyarray)

exp <- trans_exp(CESC,mrna_only = T)

write.table(exp,"CESC-GTEX-154.csv",sep = ",",row.names= T)

rm(list = ls());gc()

CESC <- read.table("CESC-DEGs.csv",sep = ",",header = T,row.names = 1,check.names = F)

View(CESC)

library(limma)

library(dplyr)

library(tidyverse)

list <- c(rep("Tumor",305),rep("Normal",10)) %>%

factor(., levels = c("Tumor", "Normal"), ordered = F)

list

list <- model.matrix(~factor(list)+0)

list

colnames(list) <- c("Tumor", "Normal")

list

df.fit <- lmFit(CESC, list)

df.fit

df.matrix <- makeContrasts(Tumor - Normal, levels = list)

df.matrix

fit <- contrasts.fit(df.fit, df.matrix)

fit <- eBayes(fit)

tempOutput <- topTable(fit,n = Inf, adjust = "BH")

tempOutput$gene <- rownames(tempOutput)

write.table(tempOutput,"TumorvsNormal.csv",

sep = ",",row.names= F)

rm(list = ls());gc()

CESC <- read.table("CESC-C.csv",sep = ",",header = T,row.names = 1,check.names = F)

View(CESC)

library(limma)

library(dplyr)

library(tidyverse)

list <- c(rep("YES",144),rep("NO",56)) %>%

factor(., levels = c("YES", "NO"), ordered = F)

list

list <- model.matrix(~factor(list)+0)

list

colnames(list) <- c("YES", "NO")

list

df.fit <- lmFit(CESC, list)

df.fit

df.matrix <- makeContrasts(YES - NO, levels = list)

df.matrix

fit <- contrasts.fit(df.fit, df.matrix)

fit <- eBayes(fit)

tempOutput <- topTable(fit,n = Inf, adjust = "BH")

tempOutput$gene <- rownames(tempOutput)

write.table(tempOutput,"YESvsNO.csv",

sep = ",",row.names= F)

BiocManager::install("org.Hs.eg.db")

rm(list = ls());gc()

#

library(stringr)

library(enrichplot)#GO,KEGG,GSEA

library(clusterProfiler)#GO,KEGG,GSEA

GO_database <- 'org.Hs.eg.db'

#

setwd("D:\\Desktop Files\\TCGA\\DEGs")

dir <- list.files()

dir

LumA <- read.table(file.choose(),sep = ",",header = F)

gene <- LumA[,1]

gene <- bitr(gene,

fromType = 'SYMBOL',

toType = 'ENTREZID',

OrgDb = GO_database)

GO <-enrichGO(gene$ENTREZID,

OrgDb = GO_database,

keyType = "ENTREZID"

ont = "BP"

pvalueCutoff = 0.05

qvalueCutoff = 0.05

readable = T)

result <- GO@result

result <- as.data.frame(result)

write.table(result,"fenxi0.5.csv",sep = ",",row.names= F)

library(data.table)

data <- fread("TCGA-CESC.htseq_counts.tsv",

sep = "\t",header = T)

write.table(data,"CESC-TCGA.csv",sep = ",",row.names= F)

rm(list = ls());gc()

library(tinyarray)

CESC <- read.table("CESC-TCGA.csv",sep = ",",header = T,row.names = 1,check.names = F)

CESC <- trans_exp(CESC,mrna_only = T)

CESC$sample <- rownames(CESC)

write.table(CESC,"CESC-TCGA.csv",sep = ",",row.names= F)

rm(list = ls());gc()

CESC <- read.table("yes.csv",sep = ",",header = T,check.names = F,row.names = 1)

t <- t(CESC)

t <- as.data.frame(t)

t$sample <- rownames(t)

gene <- read.table("40.csv",sep = ",",header = F,check.names = F)

gene <- gene[,1]

library(tidyverse)

expression <- dplyr::select(t,gene)

OS <- read.table("TCGA-CESC.survival.csv",sep = ",",header = T,check.names = F)

merge <- merge(OS,expression,by = "sample")

write.table(merge,"CESC-40-scRNA.csv",sep = ",",row.names= F)

rm(list = ls());gc()

library(survival)

library(survminer)

library(tidyverse)

library(rms)

CESC <- read.table("CESC-40-scRNA.csv",sep = ",",header = T,check.names = F,row.names = 1)

CESC <- filter(CESC,time != "0")

covariates <- colnames(CESC)[3:101]

univ_formulas <- sapply(covariates,

function(x) as.formula(paste('Surv(time, status)~', x)))

univ_models <- lapply(univ_formulas, function(x){coxph(x, data = CESC)})

univ_results <- lapply(univ_models,

function(x){

x <- summary(x)

p.value<-signif(x$wald["pvalue"], digits=4)

wald.test<-signif(x$wald["test"], digits=4)

beta<-signif(x$coef[1], digits=4);

HR <-signif(x$coef[2], digits=4);

HR.confint.lower <- signif(x$conf.int[,"lower .95"],4)

HR.confint.upper <- signif(x$conf.int[,"upper .95"],4)

HR <- paste0(HR, " (",

HR.confint.lower, "-", HR.confint.upper, ")")

res<-c(p.value,HR)

names(res)<-c("p.value","HR (95% CI for HR)")

return(res)

})

res <- t(as.data.frame(univ_results, check.names = F))

aaa <- as.data.frame(res)

write.table(file="univ_cox_result40-scRNA.csv",aaa,quote=F,sep=",")

senlin <- read.table(file.choose(),sep = ",",header = T,check.names = F)

library(forestplot)

senlin1 <- senlin[,1:3]

senlin2 <- senlin[,4:6]

forestplot(senlin1,

graph.pos = 3,

mean = senlin2$mean,

lower = senlin2$low,

upper = senlin2$high,

xticks= c(0.5,1,4),

lwd.xaxis = 3,

zero = 1,

lwd.zero = 2,

boxsize = .15,

lineheight = unit(7,'mm'),

graphwidth = unit(.2,"npc"),

colgap = unit(2,"mm"),

hrzl_lines = list("2" = gpar(lwd=2),

"8" = gpar(lwd=2, columns=1:4)),

col=fpColors(box="#1c61b6", lines="#1c61b6"),

ci.vertices=TRUE,

ci.vertices.height = 0.1,

txt_gp=fpTxtGp(label=gpar(cex=1)

ticks=gpar(cex=1)

xlab=gpar(cex=1)

title=gpar(cex=1))

)

rm(list = ls());gc()

CESC <- read.table("CESC-40-scRNA.csv",sep = ",",header = T,check.names = F)

gene <- read.table("8.csv",sep = ",",header = F,check.names = F)

gene <- gene[,1]

library(tidyverse)

expression <- dplyr::select(CESC,gene)

write.table(expression,"CESC-8-scRNA.csv",sep = ",",row.names= F)

rm(list = ls());gc()

library(foreign)

library(glmnet)

library(tidyverse)

merge <- read.table("CESC-8-scRNA.csv",sep = ",",header = T,check.names = F)

merge <- filter(merge,time != "0")

x <- as.matrix(merge[,c(4:11)])

y <- data.matrix(Surv(merge$time,merge$status))

fit<-glmnet(x,y,alpha = 1,family = "cox")

print(fit)

plot(fit,xvar = "lambda",label = T)

cvfit <- cv.glmnet(x,y,alpha=1,family="cox")

plot(cvfit)

print(cvfit)

coef.min <- coef(cvfit$glmnet.fit,s=cvfit$lambda.min,exact = F)

coef.1se <- coef(cvfit$glmnet.fit,s=cvfit$lambda.1se,exact = F)

coef.min

rm(list = ls());gc()

CESC <- read.table("CESC-6-scRNA.csv",sep = ",",header = T,check.names = F,row.names = 1)

CESC <- filter(CESC,time != "0")

res.cox <- coxph(Surv(time, status) ~ MPP5+SNX7+LSM12+VANGL1,

data=CESC)

x <- summary(res.cox)

x

pvalue = signif(as.matrix(x$coefficients)[,5],4)

HR = signif(as.matrix(x$coefficients)[,2],4)

low = signif(x$conf.int[,3],4)

high = signif(x$conf.int[,4],4)

multi_res=data.frame(p.value = pvalue,

HR = paste(HR," (",low,"-",high,")",sep=""),

HR = HR,

stringsAsFactors = F)

multi_res

write.table(file="multivariate_cox_result.csv",multi_res,quote=F,sep=",")

df <- read.table(file.choose(),header=T,sep=",",check.names = F)

ggplot(df,

aes(y = Multi_Cox.Coeficient,

x = reorder(gene,Multi_Cox.Coeficient))) +

geom_col(fill="lightblue")+

coord_flip()+

theme_bw()+

theme(legend.position = "top")+

theme(legend.title=element_blank())+

labs(x = "Gene")

library(ggrisk)

exp <- read.csv("CESC-TCGA.csv",header = F,check.names = F,row.names = 1)

t <- t(exp)

group <- read.csv("Group.csv",header = T,check.names = F)

merge <- merge(group,t,by = "sample")

merge <- merge %>% arrange(Group)

tmerge <- t(merge)

tmerge <- tmerge[-1,]

write.csv(tmerge,"zu.csv",row.names = T)

library(tidyr)

library(tidyverse)

library(survival)

library(survminer)

survival <- read.table("CESC-score.csv",header = T,sep = ",",row.names = 1)

survival$OS.time <- survival$OS.time/365

group <- ifelse(survival[,6] > quantile(survival[,6],.5),

"high","low")

diff = survdiff(Surv(OS.time,OS)~ group,data = survival)

p = 1-pchisq(diff$chisq,df = 1)

fit <- survfit(Surv(OS.time,OS)~ group,data = survival)

ggsurvplot(fit,

data = survival,

pval = p,

surv.median.line = "hv",

pval.size = 5,

legend = c(0.8,0.65),

legend.title = "Expression",

xlab = "Time(year)",

ylab = "Percent survival",

conf.int = F,

risk.table = F,

risk.table.height =.25,

break.time.by = 2,

xlim = c(0, 14))

survival <- read.table("score.csv",header = T,sep = ",",row.names = 1)

survival$time <- survival$time/365

ggplot(survival,

aes(x = number,

y = score,

color = Group)) +

geom_point() +

scale_color_manual(values = c("#f47c7c", "#1e56a0")) +

labs(x = "Patients number",

y = "Risk score") +

geom_vline(aes(xintercept=72.5), colour="grey", linetype="dashed") +

theme_classic()

ggplot(survival,

aes(x = number,

y = time,

color = Status)) +

geom_point() +

scale_color_manual(values = c("black", "red")) +

labs(x = "Patients number",

y = "Time") +

geom_vline(aes(xintercept=72.5), colour="grey", linetype="dashed") +

theme_classic()

df <- read.table(file.choose(),sep = ",",header = T,check.names = F,row.names = 1)

df1 <- t(df)

annotation_col <- as.data.frame(df1[1,])

rownames(annotation_col) <- colnames(df1)

colnames(annotation_col)[1] <- "Group"

rownames(annotation_col) <- Replace(rownames(annotation_col),from="-",to=".")

df1 <- df1[-1,]

df2 <- data.frame(df1,stringsAsFactors = F)

df2 = as.data.frame(lapply(df2,as.numeric))

rownames(df2) <- rownames(df1)

ann_colors = list(

Group = c(High="#f47c7c", Low="#1e56a0"))

library(pheatmap)

pheatmap(df2,

show_rownames = T,

show_colnames = F,

cluster_cols = F,

cluster_rows = T,

cellheight = 80,

scale = "row",

annotation_col = annotation_col,

annotation_colors = ann_colors,

border = T,

border_color = "grey",

color = colorRampPalette(c("navy", "white","red"))(100))

library(tidyverse)

library(ggpubr)

rm(list = ls());gc()

CESC <- read.table("CESC-score.csv",sep = ",",header = T,check.names = F)

outcome <- read.table("outcome.csv",header = T,sep = ",")

merge <- merge(outcome,CESC,by = "sample")

table(merge$Outcome)

gather <- gather(merge,gene,expression,5:8)

gather$expression <- as.numeric(gather$expression)

ggplot(gather,

aes(x = gene,

y = expression,

fill = Group))+

stat_compare_means(aes(group=Group),

method="t.test",

label = "p.signif",

label.y = 20,

symnum.args = list(cutpoints=c(0,0.001,0.01,0.05,1),

symbols = c("***","**","*","ns")))+

stat_boxplot(geom ='errorbar', width = 0.35,position = position_dodge(1))+

geom_boxplot(position = position_dodge(1),

outlier.size = .01,

outlier.colour = "white")+

theme_bw()+

scale_fill_manual(values = c("#f47c7c", "#1e56a0"))+

labs(x = NULL,

y = "Expression")

ggplot(gather,

aes(x = gene,

y = expression,

fill = Outcome))+

stat_compare_means(aes(group=Outcome),

method="t.test",

label = "p.signif",

label.y = 20,

symnum.args = list(cutpoints=c(0,0.001,0.01,0.05,1),

symbols = c("***","**","*","ns")))+

stat_boxplot(geom ='errorbar', width = 0.35,position = position_dodge(1))+

geom_boxplot(position = position_dodge(1),

outlier.size = .01,

outlier.colour = "white")+

theme_bw()+

scale_fill_manual(values = c("#f47c7c", "#1e56a0"))+

labs(x = NULL,

y = "Expression")

library(timeROC)

library(survival)

CESC <- read.table("CESC-score.csv",sep = ",",header = T,check.names = F)

CESC$OS.time <- CESC$OS.time/365

ROC.bili.marginal <- timeROC(T = CESC$OS.time,

delta = CESC$OS,

marker = CESC$score,

cause = 1,

weighting = "marginal",

times = c(1,2,3,5),

iid = TRUE,

ROC = T)

ROC.bili.marginal

plot(ROC.bili.marginal,time=1,title = FALSE)

plot(ROC.bili.marginal,time=2,add=TRUE,col="blue")

plot(ROC.bili.marginal,time=3,add=TRUE,col="orange")

plot(ROC.bili.marginal,time=5,add=TRUE,col="green")

legend("bottomright",y.intersp = 0.2,

c("1 year:0.51",

"2 year:0.64",

"3 year:0.75",

"5 year:0.66"),

col=c("red", "blue", "orange","green"),

lty=1, lwd=1,bty = "n")

library(rms)

CESC$OS.time <- CESC$OS.time*365

f1<-cph(formula = Surv(OS.time,OS) ~score,

data=CESC,

x=T,

y=T,

surv = T,

na.action=na.delete,

time.inc = 365)

cal1 <-calibrate(f1, cmethod="KM", method="boot",u=365,m=2500,B=1000)

f2<-cph(formula = Surv(OS.time,OS) ~score,

data=CESC,

x=T,

y=T,

surv = T,

na.action=na.delete,

time.inc = 730)

cal2 <-calibrate(f2, cmethod="KM", method="boot",u=730,m=2500,B=1000)

f3<-cph(formula = Surv(OS.time,OS) ~score,

data=CESC,

x=T,

y=T,

surv = T,

na.action=na.delete,

time.inc = 1095)

cal3 <-calibrate(f3, cmethod="KM", method="boot",u=1095,m=2500,B=1000)

f5<-cph(formula = Surv(OS.time,OS) ~score,

data=CESC,

x=T,

y=T,

surv = T,

na.action=na.delete,

time.inc = 1825)

cal5 <-calibrate(f5, cmethod="KM", method="boot",u=1825,m=2500,B=1000)

plot(cal1,

lwd = 2

lty = 1,

errbar.col = c("#2166AC")

xlim = c(0,1),ylim= c(0,1),

xlab = "Prediced OS (%)",

ylab = "Observed OS (%)",

cex.lab=1.2, cex.axis=1, cex.main=1.2, cex.sub=0.6)

plot(cal2,

lwd = 2

lty = 1

errbar.col = c("#808080")

xlim = c(0,1),ylim= c(0,1),

xlab = "Prediced OS (%)",

ylab = "Observed OS (%)",

cex.lab=1.2, cex.axis=1, cex.main=1.2, cex.sub=0.6)

par(new = TRUE)

plot(cal3,

lwd = 2

lty = 1

errbar.col = c("#3CB371")

xlim = c(0,1),ylim= c(0,1),

xlab = "Prediced OS (%)",

ylab = "Observed OS (%)",

cex.lab=1.2, cex.axis=1, cex.main=1.2, cex.sub=0.6)

par(new = TRUE)

plot(cal5,

lwd = 2

lty = 1

errbar.col = c("#DC143C"),

xlim = c(0,1),ylim= c(0,1),

xlab = "Prediced OS (%)",

ylab = "Observed OS (%)",

cex.lab=1.2, cex.axis=1, cex.main=1.2, cex.sub=0.6)

lines(cal1[,c('mean.predicted',"KM")],

type = 'b'

lwd = 2

pch = 16

col = c("#2166AC"))

box(lwd = 1)

abline(0,1,lty = 3

lwd = 2

col = c("#224444")

)

legend("topleft"

legend = c("1-year","2-year","3-year","5-year"),

col =c("#2166AC","#808080","#3CB371","#DC143C"),

lwd = 2

cex = 1.2

bty = "n")

rm(list = ls());gc()

#

library(stringr)

library(enrichplot)#GO,KEGG,GSEA

library(clusterProfiler)#GO,KEGG,GSEA

GO_database <- 'org.Hs.eg.db'

#

setwd("D:\\Desktop Files\\TCGA\\DEGs")

dir <- list.files()

dir

LumA <- read.table(file.choose(),sep = ",",header = T)

gene <- LumA[,1]

gene <- bitr(gene,

fromType = 'SYMBOL',

toType = 'ENTREZID',

OrgDb = GO_database)

GO <-enrichGO(gene$ENTREZID

OrgDb = GO_database,

keyType = "ENTREZID"

ont = "BP"

pvalueCutoff = 0.05

qvalueCutoff = 0.05

readable = T)

result <- GO@result

result <- as.data.frame(result)

write.table(result,"1up.csv",sep = ",",row.names = F)

jr <- read.table(file.choose(),sep = ",",header = T,check.names = F)

CESC <- read.table(file.choose(),sep = ",",header = T,check.names = F)

merge <- merge(CESC,jr,by = "sample")

write.table(file="merge.csv",merge,sep=",")

imu.names <- c()

cor <- c()

pvalue <- c()

for (i in 10:31) {

i1 = colnames(merge)[i]

cor_r = cor(as.numeric(merge[,8]),

as.numeric(merge[,i]),

method = "pearson")

p = cor.test(as.numeric(merge[,8]),

as.numeric(merge[,i]),

method = "pearson")[[3]]

imu.names = c(imu.names,i1)

cor = c(cor,cor_r)

pvalue = c(pvalue,p)

}

data_cor <- data.frame(imu.names,cor,pvalue)

write.table(file="cor.csv",data_cor,sep=",")

library(tidyverse)

library(ggplot2)

jinrun <- read.table(file.choose(),sep = ",",header = T,check.names = F)

jinrun1 <- jinrun[,c(1,10:31)]

jinrun2 <- gather(jinrun1,cell,value,2:23)

jinrun2 <- jinrun2[,c(1,4:6)]

ggplot(jinrun2,

aes(x=sample,

fill=cell,

y=value*100))+

geom_bar(stat = "identity", width =1)+

labs(x='Samples', y='Relative Abundance (%)')+

scale_y_continuous(expand=c(0, 0))+

theme(axis.ticks.x = element_blank(),

axis.text.x = element_blank())+

guides(colour=guide_legend(title=""))+

guides(fill = guide_legend( ncol = 1, byrow = TRUE))

install.packages("ggcorrplot")

library(ggcorrplot)

jinrun3 <- jinrun1[,2:23]

r <- cor(jinrun3, use="complete.obs")

round(r,2)

ggcorrplot(r,hc.order = TRUE)

library(ggpubr)

jinrun1$sample<- substring(jinrun1$sample,1,15)

risk <- read.table(file.choose(),sep = ",",header = T,check.names = F)

cor <- jinrun[,c(1,9:31)]

cor1 <- gather(cor,cell,value,3:24)

ggplot(cor1,

aes(x = cell,

y = value*100,

fill = Group))+

stat_compare_means(aes(group=Group),

method="t.test",

label = "p.signif",

label.y = 34,

symnum.args = list(cutpoints=c(0,0.001,0.01,0.05,1),

symbols = c("***","**","*","")))+

stat_boxplot(geom ='errorbar', width = 0.35,position = position_dodge(1))+

geom_boxplot(position = position_dodge(1),

outlier.size = .01,

outlier.colour = "white")+

theme_bw()+

scale_y_continuous(limits = c(0, 35))+

scale_fill_manual(values = c("#f47c7c", "#1e56a0"))+

labs(x = NULL,

y = "Relative Abundance(%)")+

theme(axis.text.x=element_text(angle=45,hjust=1))

jinrun <- read.table(file.choose(),sep = ",",header = T,check.names = F)

jinrun1 <- jinrun[,c(4:8,10:31)]

gene.names <- c()

cell.names <- c()

cor <- c()

pvalue <- c()

for (h in 1:5) {

h1 = colnames(jinrun1)[h]

for (i in 6:27) {

i1 = colnames(jinrun1)[i]

cor_r = cor(as.numeric(jinrun1[,h]),

as.numeric(jinrun1[,i]),

method = "pearson")

p = cor.test(as.numeric(jinrun1[,h]),

as.numeric(jinrun1[,i]),

method = "pearson")[[3]]

gene.names = c(gene.names,h1)

cell.names = c(cell.names,i1)

cor = c(cor,cor_r)

pvalue = c(pvalue,p)

}

}

data_cor <- data.frame(gene.names,cell.names,cor,pvalue)

write.table(file="cell.csv",data_cor,sep=",")

jr <- read.table("cell.csv",sep = ",",header = T,check.names = F)

jr$gene.names <- factor(jr$gene.names,levels = c("Score","GALNT3","LSM12","SNX7","MPP5"))

library(ggplot2)

ggplot(jr)+

geom_point(aes(x = cell.names,

y = gene.names,

size = cor,

fill = cor),

shape = 21)+

labs(x = NULL,

y = NULL,

color = "Correlation",

shape = "Year") +

scale_fill_gradient2(low = "blue",

mid = "white",

high = "red",

midpoint = 0,

breaks=c(0.4,0.2,0,-0.2,-0.4),

labels=c("0.4","0.2","0","-0.2","0.4"),

limits=c(-0.4,0.4))+

theme_bw()+

theme(legend.position = "top",

axis.text.x = element_text(size = 9,

face = "bold",

angle=45,hjust=1),

axis.text.y = element_text(size = 12,

face = "bold"))

+

theme(axis.text.x=element_text(angle=45,hjust=1))

rm(list = ls());gc()

library(tinyarray)

library(tidyverse)

library(dplyr)

dir <- list.files()

dir

expr <- read.table("CESC-TCGA.csv",sep = ",",header = T,check.names = F)

rownames(expr) <- expr$sample

expr <- expr[,-1]

expr_mrna <- trans_exp(expr,mrna_only = T)

t <- t(expr)

sample <- row.names(t)

expression <- cbind(sample,t)

expression <- as.data.frame.array(expression)

expression <- expression[order(expression$sample,decreasing = T),]

jcd <- dplyr::select(expression,c("PDCD1","CD274","PDCD1LG2","CTLA4","HAVCR2","LAG3",

"VTCN1","LMTK3","IDO1","TIGIT","CD27","CD28","CD40",

"CD70","CD80","CD86","CD276"))

write.table(jcd,"point.csv",

sep = ",",row.names= T)

jcd <- read.table("point.csv",sep = ",",header = T,check.names = F)

score <- read.table("score.csv",sep = ",",header = T,check.names = F)

merge <- merge(jcd,score,by = "sample")

write.table(merge,"point.csv",

sep = ",",row.names= F)

#1B3

df <- read.table(file.choose(),sep = ",",header = T,check.names = F,row.names = 1)

df1 <- t(df)

annotation_col <- as.data.frame(df[1,])

library(do)

colnames(annotation_col) <- Replace(colnames(annotation_col),from="-",to=".")

annotation_col <- t(annotation_col)

annotation_col <- as.data.frame(annotation_col)

df1 <- df[-1,]

df2 <- data.frame(df1,stringsAsFactors = F)

df2 = as.data.frame(lapply(df2,as.numeric))

rownames(df2) <- rownames(df1)

ann_colors = list(

Group = c(High="#f47c7c", Low="#1e56a0"))

library(pheatmap)

pheatmap(df2,

show_rownames = T,

show_colnames = F,

cluster_cols = T,

cluster_rows = T,

cellheight = 50,

scale = "row",

annotation_col = annotation_col,

annotation_colors = ann_colors,

border = T,

color = colorRampPalette(c("navy", "white","red"))(100))

library(ggpubr)

jinrun <- read.table(file.choose(),sep = ",",header = T,check.names = F)

cor <- jinrun[,c(1:12)]

cor1 <- gather(cor,cell,value,3:12)

ggplot(cor1,

aes(x = cell,

y = value,

fill = Group))+

stat_compare_means(aes(group=Group),

method="t.test",

label = "p.signif",

label.y = 17,

symnum.args = list(cutpoints=c(0,0.001,0.01,0.05,1),

symbols = c("***","**","*","")))+

stat_boxplot(geom ='errorbar', width = 0.35,position = position_dodge(1))+

geom_boxplot(position = position_dodge(1),

outlier.size = .01,

outlier.colour = "white")+

theme_bw()+

scale_y_continuous(limits = c(0, 17))+

scale_fill_manual(values = c("#f47c7c", "#1e56a0"))+

labs(x = NULL,

y = "Relative Abundance(%)")+

theme(axis.text.x=element_text(angle=45,hjust=1))

BiocManager::install("sva")

install.packages("GenomicFeatures")

library(oncoPredict)

library(data.table)

library(gtools)

library(reshape2)

library(ggpubr)

library(TCGAbiolinks)

library(dplyr)

library(tidyverse)

library(reshape2)

library(ggpubr)

CESC.count = fread("TCGA-CESC.htseq_counts.tsv.gz",header = T, sep = '\t',data.table = F)

CESC.pro = fread("gencode.v22.annotation.gene.probeMap",header = T, sep = '\t',data.table = F)

CESC.pro = CESC.pro[, c(1,2)]

CESC.count.pro = merge(x = CESC.pro, y = CESC.count, by.y = "Ensembl_ID", by.x = "id" )

dim(CESC.count.pro)

CESC.count.pro = distinct(CESC.count.pro,gene,.keep_all = T)

dim(CESC.count.pro)

CESC.count.pro <- column_to_rownames(CESC.count.pro, "gene")

CESC.count.pro = CESC.count.pro[,-1]

CESC.count.pro[1:4, 1:4]

yes <- read.table("sample.csv",sep = ",",header = T,check.names = F)

sample <- yes[,1]

CESC.count.pro <- dplyr::select(CESC.count.pro,sample)

rm(list = ls())

options(stringsAsFactors = F)

library(oncoPredict)

library(data.table)

library(gtools)

library(reshape2)

library(ggpubr)

dir <- "D:\\Download\\DataFiles\\Training Data"

th=theme(axis.text.x = element_text(angle = 45,vjust = 0.5))

GDSC2_Expr = readRDS(file=file.path(dir,'GDSC2_Expr (RMA Normalized and Log Transformed).rds'))

cervix <- c("COSMIC_687505","COSMIC_687506",

"COSMIC_924107","COSMIC_906824","COSMIC_906843",

"COSMIC_1298134","COSMIC_907068","COSMIC_687514",

"COSMIC_1240179","COSMIC_930297","COSMIC_1240212",

"COSMIC_930298","COSMIC_724839","COSMIC_949154")

GDSC2_Expr <- GDSC2_Expr[,colnames(GDSC2_Expr) %in% cervix]

GDSC2_Res = readRDS(file = file.path(dir,"GDSC2_Res.rds"))

GDSC2_Res <- exp(GDSC2_Res)

GDSC2_Res <- GDSC2_Res[rownames(GDSC2_Res) %in% c("COSMIC_687505","COSMIC_687506",

"COSMIC_924107","COSMIC_906824","COSMIC_906843",

"COSMIC_1298134","COSMIC_907068","COSMIC_687514",

"COSMIC_1240179","COSMIC_930297","COSMIC_1240212",

"COSMIC_930298","COSMIC_724839","COSMIC_949154"), ]

GDSC2_Res <- as.matrix(GDSC2_Res)

GDSC2_Res <- GDSC2_Res[ , colSums(is.na (GDSC2_Res))== 0 ]

testExpr <- CESC.count.pro[,sample(1:ncol(CESC.count.pro),144)]

testExpr <- as.matrix(testExpr)

testExpr[1:4,1:4]

dim(testExpr)

calcPhenotype(trainingExprData = GDSC2_Expr,

trainingPtype = GDSC2_Res,

testExprData = testExpr,

batchCorrect = 'eb',

powerTransformPhenotype = TRUE,

removeLowVaryingGenes = 0.2,

minNumSamples = 20,

printOutput = TRUE,

removeLowVaringGenesFrom = 'homogenizeData' )

result <- read.csv("DrugPredictions.csv", header = T , stringsAsFactors = F ,check.names = F)

result[1:4, 1:4]

gene_drug <- CESC.count.pro %>%

t() %>%

as.data.frame() %>%

dplyr::select(c("MPP5","SNX7","LSM12","GALNT3")) %>%

rownames_to_column("sample") %>%

inner_join(result)

write.table(gene_drug,"gene_drug.csv",

sep = ",",row.names= F)

score <- read.table("CESC-score.csv",sep = ",",header = T,check.names = F)

merge <- merge(score,gene_drug,by = "sample")

write.table(merge,"gene_drug.csv",

sep = ",",row.names= F)

gene_drug <- read.table("gene_drug.csv",sep = ",",header = T,check.names = F)

gene.names <- c()

drug.names <- c()

cor <- c()

pvalue <- c()

for (h in 2:5) {

h1 = colnames(gene_drug)[h]

for (i in 6:182) {

i1 = colnames(gene_drug)[i]

cor_r = cor(as.numeric(gene_drug[,h]),

as.numeric(gene_drug[,i]),

method = "pearson")

p = cor.test(as.numeric(gene_drug[,h]),

as.numeric(gene_drug[,i]),

method = "pearson")[[3]]

gene.names = c(gene.names,h1)

drug.names = c(drug.names,i1)

cor = c(cor,cor_r)

pvalue = c(pvalue,p)

}

}

data_cor <- data.frame(gene.names,drug.names,cor,pvalue)

write.table(file="drug.csv",data_cor,sep=",")

merge <- read.table("gene_drug.csv",sep = ",",header = T,check.names = F)

drug.names <- c()

cor <- c()

pvalue <- c()

for (i in 8:184) {

i1 = colnames(merge)[i]

cor_r = cor(as.numeric(merge[,6]),

as.numeric(merge[,i]),

method = "pearson")

p = cor.test(as.numeric(merge[,6]),

as.numeric(merge[,i]),

method = "pearson")[[3]]

drug.names = c(drug.names,i1)

cor = c(cor,cor_r)

pvalue = c(pvalue,p)

}

data_cor <- data.frame(drug.names,cor,pvalue)

write.table(file="drug-group.csv",data_cor,sep=",")

setwd("E:\\desktop\\else\\CC\\data")

library(tidyr)

result <- read.csv("gene_drug.csv", header = T , stringsAsFactors = F ,check.names = F)

result1 <- result[,colnames(result) %in%

c("sample","MPP5","SNX7","LSM12","GALNT3","score","Group",

"Cisplatin_1005","Docetaxel_1007","Paclitaxel_1080",

"Oxaliplatin_1089","Oxaliplatin_1806","Sepantronium bromide_1941",

"Talazoparib_1259","AGI-5198_1913","Savolitinib_1936",

"Pevonedistat_1529","Gemcitabine_1190")]

library(ggstatsplot)

#1 Docetaxel_1007

ggscatterstats(

data = result1,

x = score,

y = Docetaxel_1007,

xlab = "Score",

marginal = TRUE,

marginal.type = "densigram",

margins = "both",

xfill = "blue",

yfill = "#009E73",

title = "Relationship between Score and Docetaxel_1007",

messages = FALSE

)

#2 Cisplatin_1005

ggscatterstats(

data = result1,

x = score,

y = Cisplatin_1005,

xlab = "Score",

marginal = TRUE,

marginal.type = "densigram",

margins = "both",

xfill = "blue",

yfill = "#009E73",

title = "Relationship between Score and Cisplatin_1005",

messages = FALSE

)

#3 Paclitaxel_1080

ggscatterstats(

data = result1,

x = score,

y = Paclitaxel_1080,

xlab = "Score",

marginal = TRUE,

marginal.type = "densigram",

margins = "both",

xfill = "blue",

yfill = "#009E73",

title = "Relationship between Score and Paclitaxel_1080",

messages = FALSE

)

#4 Oxaliplatin_1089

ggscatterstats(

data = result1,

x = score,

y = Oxaliplatin_1089,

xlab = "Score",

marginal = TRUE,

marginal.type = "densigram",

margins = "both",

xfill = "blue",

yfill = "#009E73",

title = "Relationship between Score and Oxaliplatin_1089",

messages = FALSE

)

#5 Oxaliplatin_1806

ggscatterstats(

data = result1,

x = score,

y = Oxaliplatin_1806,

xlab = "Score",

marginal = TRUE,

marginal.type = "densigram",

margins = "both",

xfill = "blue",

yfill = "#009E73",

title = "Relationship between Score and Oxaliplatin_1806",

messages = FALSE

)

#6 Sepantronium bromide_1941

ggscatterstats(

data = result1,

x = score,

y = "Sepantronium bromide_1941",

xlab = "Score",

marginal = TRUE,

marginal.type = "densigram",

margins = "both",

xfill = "blue",

yfill = "#009E73",

title = "Relationship between Score and Sepantronium bromide_1941",

messages = FALSE

)

#7 Talazoparib_1259

ggscatterstats(

data = result1,

x = score,

y = Talazoparib_1259,

xlab = "Score",

marginal = TRUE,

marginal.type = "densigram",

margins = "both",

xfill = "blue",

yfill = "#009E73",

title = "Relationship between Score and Talazoparib_1259",

messages = FALSE

)

#8 AGI-5198_1913

ggscatterstats(

data = result1,

x = score,

y = "AGI-5198_1913",

xlab = "Score",

marginal = TRUE,

marginal.type = "densigram",

margins = "both",

xfill = "blue",

yfill = "#009E73",

title = "Relationship between Score and AGI-5198_1913",

messages = FALSE

)

#9 Savolitinib_1936

ggscatterstats(

data = result1,

x = score,

y = Savolitinib_1936,

xlab = "Score",

marginal = TRUE,

marginal.type = "densigram",

margins = "both",

xfill = "blue",

yfill = "#009E73",

title = "Relationship between Score and Savolitinib_1936",

messages = FALSE

)

#10 Pevonedistat_1529

ggscatterstats(

data = result1,

x = score,

y = Pevonedistat_1529,

xlab = "Score",

marginal = TRUE,

marginal.type = "densigram",

margins = "both",

xfill = "blue",

yfill = "#009E73",

title = "Relationship between Score and Pevonedistat_1529",

messages = FALSE

)

#11 Gemcitabine_1190

ggscatterstats(

data = result1,

x = score,

y = Gemcitabine_1190,

xlab = "Score",

marginal = TRUE,

marginal.type = "densigram",

margins = "both",

xfill = "blue",

yfill = "#009E73",

title = "Relationship between Score and Gemcitabine_1190",

messages = FALSE

)

library(ggplot2)

library(ggpubr)

for (i in 8:18) {

p <- ggplot(result1,

aes(x = Group,

y = result1[[i]],

fill = Group))+

stat_boxplot(geom ="errorbar",width=0.5,position=position_dodge(0.75),)+

geom_boxplot(outlier.shape = NA)+

scale_fill_manual(values = c("#f47c7c", "#1e56a0"))+

stat_compare_means(aes(group = Group), label = "p.signif",size = 8,

label.x = 1.5,

symnum.args = list(cutpoints=c(0,0.001,0.01,0.05,1),

symbols = c("***","**","*","ns")))+

xlab(NULL)+

ylab(colnames(result1)[i])+

theme_bw()+

theme(text = element_text(size = 20),

legend.position = "none")

ggsave(paste0(colnames(result1)[i],".pdf"),width = 10,height = 7.5)

print(p)

dev.off()

}

library(readr)

gene_list <- read_csv("1.csv", col_names = FALSE)

target_genes <- gene_list$X1

expression_matrix <- read.csv("zu.csv",

row.names = 1

check.names = FALSE)

filtered_data <- expression_matrix[rownames(expression_matrix) %in% target_genes, ]

missing_genes <- setdiff(target_genes, rownames(filtered_data))

if (length(missing_genes) > 0) {

message("missing_genes：")

print(missing_genes)

}

head(filtered_data)

write.csv(filtered_data, "11.csv")

library(ggplot2)

data <- read.csv("filtered_expression_data.csv", row.names = 1)

data <- t(data)

data <- as.data.frame(data)

non_constant_cols <- sapply(data, function(col) var(col, na.rm = TRUE) != 0)

new_data <- data[, non_constant_cols]

group <- factor(substr(rownames(data), 1, 3))

pca_result <- prcomp(new_data, scale = TRUE)

pca_df <- as.data.frame(pca_result$x)

pca_df$Group <- group

variance <- pca_result$sdev^2 / sum(pca_result$sdev^2) * 100

my_colors <- c("Low" = "#046BBB","Hig" = "#C9403A")

ggplot(pca_df, aes(x = PC1, y = PC2, color = Group)) +

geom_vline(xintercept = 0, linetype = "dashed", color = "gray60") +

geom_hline(yintercept = 0, linetype = "dashed", color = "gray60") +

geom_point(size = 4, alpha = 0.8) +

stat_ellipse(

aes(fill = Group),

geom = "polygon",

alpha = 0.2,

level = 0.95,

show.legend = FALSE

) +

scale_color_manual(values = my_colors) +

scale_fill_manual(values = my_colors) +

labs(

x = paste0("PC1 (", round(variance[1], 1), "%)"),

y = paste0("PC2 (", round(variance[2], 1), "%)"),

title = "PCA Analysis"

) +

theme_bw(base_size = 12) +

theme(

plot.title = element_text(hjust = 0.5, face = "bold"),

panel.grid.major = element_line(color = "gray90"),

panel.grid.minor = element_blank(),

legend.position = "none"

)

expr_data <- read.csv("11.csv", row.names = 1)

sample_groups <- data.frame(

Group = factor(rep(c("High", "Low"), each = 72)),

row.names = colnames(expr_data)

)

max_abs <- max(abs(range(expr_data)))

color_breaks <- seq(-max_abs, max_abs, length.out = 20)

library(pheatmap)

pheatmap(expr_data,

color = colorRampPalette(c("navy", "white", "red"))(20),

annotation_col = sample_groups,

show_rownames = TRUE,

show_colnames = F,

cluster_rows = TRUE,

cluster_cols = F,

fontsize_row = 8,

scale = "row",

main = "Gene Expression Heatmap (High vs Low)",

annotation_colors = list(

Group = c(Low = "#046BBB", High = "#C9403A")

))

setwd("E:\\desktop")

data <- read.csv("SNX7.csv")

min_max_scale <- function(x) {

(x - min(x)) / (max(x) - min(x))

}

data$exp_scaled <- min_max_scale(data$exp)

write.csv(data, "SNX7_scaled.csv", row.names = FALSE)

no <- read.csv("NO.csv",header = T)

sur <- read.csv("TCGA-CESC.survival.csv",header = T)

merge <- merge(no,sur,by = "sample")

merge$OS.time <- merge$OS.time/365

diff = survdiff(Surv(OS.time,OS)~ Group,data = merge)

p = 1-pchisq(diff$chisq,df = 1)

fit <- survfit(Surv(OS.time,OS)~ Group,data = merge)

ggsurvplot(fit,

data = merge,

pval = p,

surv.median.line = "hv",

pval.size = 5,

legend = c(0.9,0.9),

legend.title = "Risk",

xlab = "Time(year)",

ylab = "Percent survival",

conf.int = F,

risk.table = F,

risk.table.height =.25,

break.time.by = 2,

xlim = c(0, 14))

group <- read.csv("2.csv",header = T,check.names = F)

CESC <- read.csv("CESC-TCGA.csv",header = T,check.names = F,row.names = 1)

samples_to_extract <- group$sample

existing_samples <- intersect(samples_to_extract, colnames(CESC))

extracted_data <- CESC %>%

dplyr::select(all_of(existing_samples)) %>%

as.data.frame()

missing_samples <- setdiff(samples_to_extract, colnames(CESC))

if (length(missing_samples) > 0) {

warning("以下样本在测序数据中未找到：\n", paste(missing_samples, collapse = ", "))

}

extracted_data <- extracted_data[, samples_to_extract[samples_to_extract %in% existing_samples]]

rm(list = ls())

options(stringsAsFactors = F)

library(oncoPredict)

library(data.table)

library(gtools)

library(reshape2)

library(ggpubr)

BiocManager::install("sva")

dir <- "D:\\Download\\DataFiles\\Training Data"

th=theme(axis.text.x = element_text(angle = 45,vjust = 0.5))

GDSC2_Expr = readRDS(file=file.path(dir,'GDSC2_Expr (RMA Normalized and Log Transformed).rds'))

cervix <- c("COSMIC_687505","COSMIC_687506",

"COSMIC_924107","COSMIC_906824","COSMIC_906843",

"COSMIC_1298134","COSMIC_907068","COSMIC_687514",

"COSMIC_1240179","COSMIC_930297","COSMIC_1240212",

"COSMIC_930298","COSMIC_724839","COSMIC_949154")

GDSC2_Expr <- GDSC2_Expr[,colnames(GDSC2_Expr) %in% cervix]

GDSC2_Res = readRDS(file = file.path(dir,"GDSC2_Res.rds"))

GDSC2_Res <- exp(GDSC2_Res)

GDSC2_Res <- GDSC2_Res[rownames(GDSC2_Res) %in% c("COSMIC_687505","COSMIC_687506",

"COSMIC_924107","COSMIC_906824","COSMIC_906843",

"COSMIC_1298134","COSMIC_907068","COSMIC_687514",

"COSMIC_1240179","COSMIC_930297","COSMIC_1240212",

"COSMIC_930298","COSMIC_724839","COSMIC_949154"), ]

GDSC2_Res <- as.matrix(GDSC2_Res)

GDSC2_Res <- GDSC2_Res[ , colSums(is.na (GDSC2_Res))== 0 ]

testExpr <- extracted_data[,sample(1:ncol(extracted_data),200)]

testExpr <- as.matrix(testExpr)

testExpr[1:4,1:4]

dim(testExpr)

calcPhenotype(trainingExprData = GDSC2_Expr,

trainingPtype = GDSC2_Res,

testExprData = testExpr,

batchCorrect = 'eb',

powerTransformPhenotype = TRUE,

removeLowVaryingGenes = 0.2,

minNumSamples = 20,

printOutput = TRUE,

removeLowVaringGenesFrom = 'homogenizeData' )

result <- read.csv("DrugPredictions.csv", header = T , stringsAsFactors = F ,check.names = F)

result[1:4, 1:4]

gene_drug <- CESC.count.pro %>%

t() %>%

as.data.frame() %>%

dplyr::select(c("MPP5","SNX7","LSM12","GALNT3")) %>%

rownames_to_column("sample") %>%

inner_join(result)

write.table(gene_drug,"gene_drug.csv",

sep = ",",row.names= F)

merge <- merge(group,result,by = "sample")

write.table(merge,"group_drug.csv",

sep = ",",row.names= F)

gene_drug <- read.table("group_drug.csv",sep = ",",header = T,check.names = F)

drug.names <- c()

cor <- c()

pvalue <- c()

for (i in 3:179) {

i1 = colnames(gene_drug)[i]

cor_r = cor(as.numeric(gene_drug[,2]),

as.numeric(gene_drug[,i]),

method = "pearson")

p = cor.test(as.numeric(gene_drug[,2]),

as.numeric(gene_drug[,i]),

method = "pearson")[[3]]

drug.names = c(drug.names,i1)

cor = c(cor,cor_r)

pvalue = c(pvalue,p)

}

data_cor <- data.frame(gene.names,drug.names,cor,pvalue)

write.table(file="drug.csv",data_cor,sep=",")

merge <- read.table("gene_drug.csv",sep = ",",header = T,check.names = F)

data <- read.csv("group_drug.csv", row.names = 1)

str(data)

data$group <- as.factor(data$group)

drugs <- colnames(data)[-1] # 排除分组列

results <- data.frame(

Drug = character(),

Test = character(),

W = numeric(),

p_value = numeric(),

stringsAsFactors = FALSE

)

for (drug in drugs) {

test_result <- wilcox.test(

formula = as.formula(paste(drug, "~ group")),

data = data

)

results <- rbind(results, data.frame(

Drug = drug,

p_value = test_result$p.value

))

}

write.csv(data,"药.csv")

library(tidyverse)

p5 <- filter(results,results$p_value < 0.05)

p5 <- as.data.frame(p5)

write.csv(p5,"p5.csv")

data <- read.csv("72.csv",header = T,row.names = 1)

drugs <- colnames(data)[-1]

library(ggplot2)

for (drug in drugs) {

p <- ggplot(data, aes(x = group, y = .data[[drug]], fill = group)) +

geom_boxplot() +

labs(title = paste("Distribution of", drug),

x = "Group", y = "Drug Response") +

theme_minimal()

print(p)

}

group <- read.csv("p5.csv",header = F,check.names = F)

CESC <- read.csv("CESC-TCGA.csv",header = T,check.names = F,row.names = 1)

samples_to_extract <- group$V1

existing_samples <- intersect(samples_to_extract, colnames(data))

extracted_data <- data %>%

dplyr::select(all_of(existing_samples)) %>%

as.data.frame()

write.csv(extracted_data,"72.csv")

library(ggplot2)

aa <- read.table("HighVSLow.csv",sep = ",",header = T,check.names = F)

aa$Group <- factor(aa$Group,levels = c("up","no","down"))

ggplot(aa,aes(x = logFC,y = -log10(P.Value)))+

geom_point(aes(color=Group),size = 3)+

ylab("-log10(P.Value)") +

xlab("Log2FC") +

theme_bw()+

scale_color_manual(values=c(up = "#E01010", down = "#226ED1", no = "#BEBEBE")) +

geom_vline(xintercept = c(-0.415,0.585),linetype = "dashed")+

geom_hline(yintercept = 1.3,linetype = "dashed")

library(ggplot2)

library(ggpubr)

ggplot(data,

aes(x = group,

y = Talazoparib_1259,

fill = group))+

stat_boxplot(geom ="errorbar",width=0.4,position=position_dodge(0.5))+

geom_boxplot()+

scale_fill_manual(values = c("#9DB4CE", "#F9C08A"))+

stat_compare_means(aes(group = group), label.x = 1.25,

method = "wilcox.test",size = 5)+

xlab(NULL)+

theme_bw()+

theme(text = element_text(size = 20),

legend.position = "none")
