# Supplementary figures and images for: Construction of a prognostic prediction model for concurrent radiotherapy in cervical cancer using GEO and TCGA databases with preliminary validation analysis
Source: PLoS One. 2025 Oct 31;20(10):e0334281. doi: 10.1371/journal.pone.0334281 (PMC12578209; doi:10.1371/journal.pone.0334281)

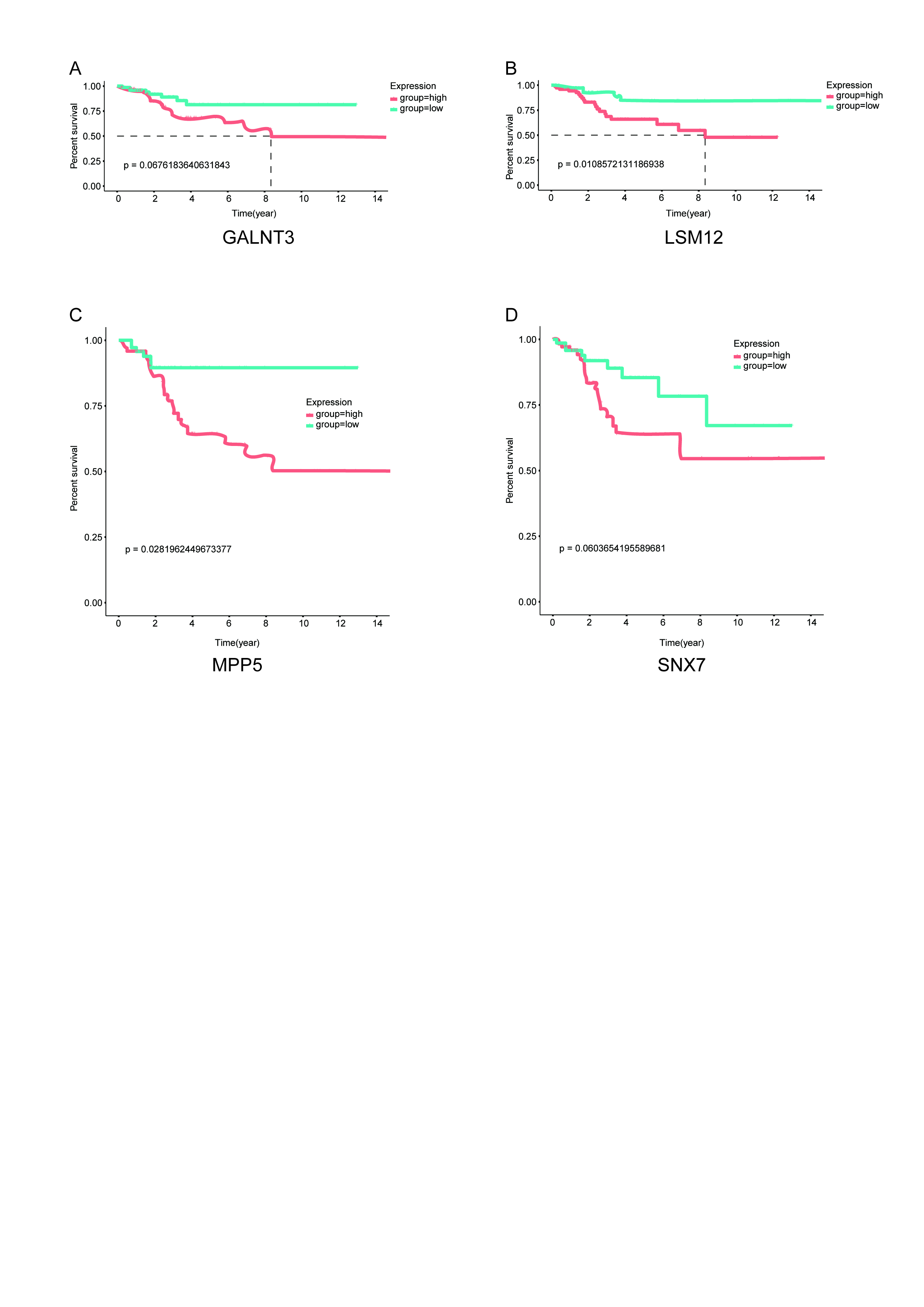

Supplement: S1 Fig — (A) GALNT3. (B) LSM12. (C) MPP5. (D)SNX7. (*p < 0.05, **p < 0.01, ***p < 0.001, ****p < 0.0001). (TIF) [file pone.0334281.s002.tif]
