# Supplementary material for: Construction of a prognostic prediction model for concurrent radiotherapy in cervical cancer using GEO and TCGA databases with preliminary validation analysis
Source: PLoS One. 2025 Oct 31;20(10):e0334281. doi: 10.1371/journal.pone.0334281 (PMC12578209; doi:10.1371/journal.pone.0334281)
Supplement: Attachment — Submitted filename: PONE-D-25-08333-ResponseLetter.docx [file pone.0334281.s004.docx]

**Summary of Changes (for the first round)**

Dear editor and anonymous reviewers,

We really want to express our appreciation for your insightful comments, constructive suggestions and valuable time for the paper. We sincerely appreciate the encouragement in your comments.

In the first round, reviewers made the following main comments on our paper:

(1) **Journal formatting and data/code sharing requirements:** The editor emphasized strict compliance with PLOS ONE’s formatting guidelines, funding information policies, and the mandatory sharing of data and code to ensure reproducibility of the study findings.
(2) **Limited short-term predictive performance of the model**
    Reviewers raised concerns about the model’s AUC of 0.51 at 1 year, which contradicts the claim of “strong predictive capabilities.” They requested that the limitations of short-term predictions be acknowledged and discussed in detail.
(3) **Accuracy of statistical interpretation and reporting**
    Reviewers noted an incorrect correlation coefficient (1.41), potentially leading to misinterpretation. They also recommended citing the TRIPOD guidelines and ensuring full disclosure of analysis code and raw IHC data.

We highly appreciate reviewer’s feedback and have taken steps to address those comments. The major modifications in this revised version are reported as follows.

(1) **Regarding journal requirements**: We have thoroughly revised the manuscript to meet PLOS ONE’s formatting standards, removed funding information from the main text, and made all analysis code and raw data (including IHC MOD values) publicly available on Figshare, in full compliance with PLOS ONE’s data sharing policy.
(2) **Regarding model performance**: We have revised the Discussion and Conclusion sections to clearly acknowledge the model’s limited short-term predictive power, emphasizing its stronger applicability in medium- to long-term follow-ups and avoiding overstatements of “strong predictive capabilities.”
(3) **Regarding statistical interpretation**: We have corrected the erroneous correlation coefficient (clarified as an IC50-related metric) and added explanations in the Methods section. The TRIPOD guidelines have also been cited to improve the quality of reporting.

We have thoroughly revised the paper based on the reviewers' suggestions and documented all changes in the revised manuscript. We believe these improvements significantly enhance the quality of the paper and look forward to the reviewers' further feedback.

**Response to editor and reviewers**

We carefully revise our paper according to the helpful comments of the reviewers and associate editor. We answer all of questions and comments one by one and revise the paper accordingly. In the revised paper, the revised sentences have been highlighted in **blue color.**

***Editor：***

*1. Please ensure that your manuscript meets PLOS ONE's style requirements, including those for file naming. The PLOS ONE style templates can be found at https://journals.plos.org/plosone/s/file?id=wjVg/PLOSOne_formatting_sample_main_body.pdf and* <https://journals.plos.org/plosone/s/file?id=ba62/PLOSOne_formatting_sample_title_authors_affiliations.pdf>

***Reply:***

Based on the editor’s comments, we have carefully revised the entire manuscript to ensure it fully complies with PLOS ONE’s formatting requirements.

*2. Please note that PLOS ONE has specific guidelines on code sharing for submissions in which author-generated code underpins the findings in the manuscript. In these cases, we expect all author-generated code to be made available without restrictions upon publication of the work. Please review our guidelines at https://journals.plos.org/plosone/s/materials-and-software-sharing#loc-sharing-code and ensure that your code is shared in a way that follows best practice and facilitates reproducibility and reuse.*

***Reply:***

We have reviewed the code sharing policy at [PLOS ONE Materials and Software Sharing – Sharing Code](https://journals.plos.org/plosone/s/materials-and-software-sharing#loc-sharing-code). In accordance with the guidelines, all author-generated code supporting the findings of this study has been made publicly available on Figshare to ensure reproducibility and reusability. The code can be accessed at: https://figshare.com/articles/dataset/data_docx/29588924

*3. Please note that funding information should not appear in any section or other areas of your manuscript. We will only publish funding information present in the Funding Statement section of the online submission form. Please remove any funding-related text from the manuscript.*

***Reply:***

We have carefully removed all funding information from the manuscript text, as per the journal’s policy. All funding details are now provided exclusively in the online submission form’s funding statement section.

*4. Thank you for stating the following financial disclosure:*

*“This study was supported by National Natural Science Foundation of China (82373207), Climing program of Harbin Medical University Cancer Hospital (PDYS2024-06).”*

*Please state what role the funders took in the study.  If the funders had no role, please state: "The funders had no role in study design, data collection and analysis, decision to publish, or preparation of the manuscript."*

*If this statement is not correct you must amend it as needed.*

*Please include this amended Role of Funder statement in your cover letter; we will change the online submission form on your behalf.*

***Reply:***

We appreciate your note regarding the funding disclosure. The funding agencies, including the National Natural Science Foundation of China (Grant No. 82373207) and the Climing Project of Harbin Medical University Cancer Hospital (PDYS2024-06), had no role in study design, data collection and analysis, decision to publish, or preparation of the manuscript. We have included this clarified funding statement in the cover letter as requested.

*5. We note that your Data Availability Statement is currently as follows: All relevant data are within the manuscript and in Supporting Information files.*

*Please confirm at this time whether or not your submission contains all raw data required to replicate the results of your study. Authors must share the “minimal data set” for their submission. PLOS defines the minimal data set to consist of the data required to replicate all study findings reported in the article, as well as related metadata and methods ([https://journals.plos.org/plosone/s/data-availability#loc-minimal-data-set-definition](https://journals.plos.org/plosone/s/data-availability#loc-minimal-data-set-definition)).*

***Reply:***

We confirm that all data necessary to replicate the results reported in this manuscript are included within the manuscript and the supporting information files, constituting the minimal data set as defined by PLOS ONE.

*6. Please review your reference list to ensure that it is complete and correct. If you have cited papers that have been retracted, please include the rationale for doing so in the manuscript text, or remove these references and replace them with relevant current references. Any changes to the reference list should be mentioned in the rebuttal letter that accompanies your revised manuscript. If you need to cite a retracted article, indicate the article’s retracted status in the References list and also include a citation and full reference for the retraction notice.*

***Reply:***

We have carefully reviewed the reference list to ensure it is complete and accurate. No retracted articles are cited in the manuscript. If any cited article had been retracted, we would have either removed it or clearly indicated its retracted status in the references along with the retraction notice, as per journal policy. Any changes to the reference list have been detailed in the accompanying response letter.

*-----------------------------------------------------------------------------------------------------------------*

***Reviewer: 1***
*This study provides insightful model of prognostic prediction for concurrent radiotherapy in cervical cancer using GEO and TCGA databases with preliminary validation analysis. This study is well-organized and written; however, it depends on selected retrospective dataset, limited experimental validity, and demographic constraints. This contributes to study limitation but it is acknowledged by authors in discussion. Overall, this study contributes to valuable foundation for future research consideration.*

***Reply:***

We sincerely thank the reviewer for the positive and constructive comments regarding our study. We appreciate your recognition of the organization and clarity of our manuscript. We also acknowledge the limitations you pointed out, including reliance on retrospective datasets, limited experimental validation, and demographic constraints. As noted, these limitations were addressed in the Discussion section to provide a balanced perspective. We agree that our study lays a valuable foundation for future research, and we will continue to explore these aspects further to strengthen the findings. Thank you again for your insightful feedback.

*-----------------------------------------------------------------------------------------------------------------*

***Reviewer: 2***

*The study addresses a clinically relevant issue — the identification of biomarkers for radiotherapy resistance in cervical cancer. The approach that integrates data from various platforms is commendable and demonstrates an effort to validate the findings in silico. However, when analyzing the technical robustness and data support for the conclusions, some areas for improvement and clarification emerge.*

*1. Statistical Analysis*

*The variation of the AUC (0.51 for 1 year, rising to 0.75 at 3 years, and dropping to 0.66 at 5 years) suggests an inconsistency in the model’s performance over time. Although 0.75 at 3 years is a reasonable value (indicating moderate to good discriminatory capacity), the starting point of 0.51 for 1 year is a red flag. A more thorough discussion of the AUCs, particularly the 1-year AUC, is needed to acknowledge its implications. The article’s conclusions state that the model demonstrated “strong predictive capabilities for patient outcomes in radiotherapy.” However, the 0.51 AUC contradicts the idea of “strong predictive capabilities” for all timeframes, especially in the short term. The authors should openly discuss this variation and the low initial performance, as well as their implications. If the model is not good at predicting outcomes in 1 year, this needs to be acknowledged as a clear limitation.
(1) The variation of the AUC (0.51 for 1 year, rising to 0.75 at 3 years, and dropping to 0.66 at 5 years) suggests an inconsistency in the model’s performance over time. Although 0.75 at 3 years is a reasonable value (indicating moderate to good discriminatory capacity), the starting point of 0.51 for 1 year is a red flag. A more thorough discussion of the AUCs, particularly the 1-year AUC, is needed to acknowledge its implications.*

***Reply:***

We thank the reviewer for pointing out the issue regarding the time-varying AUC values of our prognostic model. As stated in our study, radiotherapy tolerance in cervical cancer remains a complex clinical challenge, with its underlying molecular mechanisms not yet fully elucidated. Through an integrative analysis of GEO single-cell and bulk RNA sequencing datasets, we identified four target genes—MPP5, SNX7, LSM12, and GALNT3—and validated them using clinical samples from the TCGA database. Based on these, we developed a prognostic model to predict patient outcomes following radiotherapy.

We have explicitly acknowledged this limitation in the revised manuscript and have revised the conclusion accordingly. We now emphasize that the model shows reliable performance primarily in medium- to long-term prognosis, rather than strong predictive ability across all time points. The calibration curves further support the overall reliability of the model over time.

To avoid overstating the model’s capabilities, we have revised the conclusion to reflect that the model exhibits *relatively reliable* performance in medium- to long-term predictions, rather than demonstrating “strong” predictive ability at all time points.

**☆ Section 4: Discussion (Page 17):**

The prognostic risk model constructed in this study, based on the four genes MPP5, SNX7, LSM12, and GALNT3, demonstrated promising predictive performance for radiotherapy outcomes in cervical cancer patients, particularly in the medium- to long-term follow-up, with an AUC of 0.75 at 3 years. However, its short-term predictive ability was relatively limited, as reflected by a lower AUC of 0.51 at 1 year. This finding may indicate that early clinical outcomes following radiotherapy are influenced by a range of complex factors, including substantial heterogeneity in initial treatment responses, which may not be fully captured by baseline gene expression profiles.

Moreover, the variability in clinical data and the limited sample size may also have contributed to the reduced stability of the model’s short-term predictions. These factors highlight the challenges in developing robust short-term prognostic models and underscore the need for incorporating additional clinical indicators and real-time monitoring data in future studies to enhance predictive accuracy during the early stages of treatment.

**☆ Section 5: Conclusion (Page 21):**

The prognostic model shows greater applicability in medium- to long-term survival prediction, rather than short-term outcome estimation. Calibration curves demonstrate good predictive consistency across all time points, supporting the model’s potential for clinical application. Future studies should incorporate additional clinical variables and dynamic monitoring data to enhance the accuracy of early radiotherapy response prediction.

*(2) The article’s conclusions state that the model demonstrated “strong predictive capabilities for patient outcomes in radiotherapy.” However, the 0.51 AUC contradicts the idea of “strong predictive capabilities” for all timeframes, especially in the short term. The authors should openly discuss this variation and the low initial performance, as well as their implications. If the model is not good at predicting outcomes in 1 year, this needs to be acknowledged as a clear limitation.*
***Reply:***

We thank the reviewer for highlighting the inconsistency between our description of the model’s “strong predictive capabilities” and the relatively low AUC of 0.51 at the 1-year time point. We acknowledge that the model shows limited predictive performance in the short term, in contrast to its moderate to good discriminatory ability observed at later time points, such as an AUC of 0.75 at 3 years.

In response, we have explicitly addressed the time-dependent variation in predictive accuracy in the revised manuscript, emphasizing that the model’s strength lies primarily in medium- to long-term follow-up. The low AUC at 1 year is now clearly acknowledged as a key limitation, which likely reflects the inability of gene expression profiles to fully capture the biological and clinical complexity influencing early prognosis after radiotherapy.

Accordingly, we have revised the conclusion to more accurately state that, while the model has predictive value for radiotherapy outcomes overall, its short-term predictive power is limited. We believe these revisions provide a more balanced and transparent interpretation of our findings.

**☆ Section 3.3 (Page 14&15):**

However, the relatively low AUC at 1 year (0.51) suggests that the model has limited predictive power for short-term outcomes. This may be attributed to the complexity and heterogeneity of early treatment responses, which might not be fully captured by baseline gene expression profiles. In contrast, the model demonstrated moderate to good discriminatory performance in the medium- to long-term follow-up (e.g., AUC = 0.75 at 3 years), indicating its greater utility in longer-term prognostic assessment. Therefore, while the overall performance supports its clinical potential, the model should be interpreted with caution when applied to short-term outcome prediction.

*2. The discussion about the role of genes in immune infiltration and chemosensitivity is interesting, but the correlations presented (e.g., r=1.41 for gemcitabine — which seems like a typo, as the Pearson correlation coefficient should range from -1 to 1) need to be reviewed to ensure accuracy and correct interpretation.*

***Reply:***

We thank the reviewer for the positive feedback on our discussion regarding the role of genes in immune infiltration and chemosensitivity. We also appreciate the reviewer’s careful observation regarding the reported Pearson correlation coefficient exceeding the valid range of [-1, 1]. Upon review, we found that this was a typographical error. The reported value of 1.41 referred not to a correlation coefficient, but rather to a predicted IC50-related metric derived from the drug sensitivity analysis. We have corrected this in the revised manuscript and clarified in both the text and figure legend to distinguish correlation coefficients from other modeling outputs, thereby avoiding any potential misinterpretation.

**☆ Section 3.5 (Page 15):**

A significant negative correlation was observed between risk scores and gemcitabine concentration (Pearson coefficient: r = –0.41, p = 0.033).

*3. Full Data Availability
PLOS ONE's data policy is clear: it requires that all data underlying the findings be made fully and freely available, unless there are legal or ethical exceptions that must be specified. The study involved complex computational analyses (Cox regression, Lasso Cox regression, UMAP, CIBERSORT, oncoPredict, etc.) using R software. PLOS ONE’s policy encourages the sharing of analysis code/scripts to ensure reproducibility. Although the authors mention the use of R packages, there is no indication that the custom scripts used to generate the results (such as the risk model construction, ROC curves, immune infiltration heatmaps, and pharmacogenomic analyses) were made publicly available in a repository (e.g., GitHub, Zenodo) with a DOI. This is a critical point for the study’s reproducibility. The authors did make the public database data (GEO and TCGA) available, which is positive. However, it is unclear whether the raw data underlying the IHC analyses (such as the MOD values for each individual sample) and the custom codes/scripts used for the computational analyses were fully made available in an accessible format and in an appropriate repository, as required by PLOS ONE for complete data availability. The generic statement “All relevant data are within the manuscript and its Supporting Information files” is generally not sufficient to comply with this guideline for proprietary data and complex scripts.*

***Reply:***

We appreciate the reviewer’s detailed comments regarding data sharing and reproducibility. We fully acknowledge PLOS ONE’s policy on making all underlying data and analysis code publicly available.

The public datasets used in this study (GEO and TCGA) have been appropriately cited and their accession numbers provided within the manuscript and supporting information.

Regarding the custom R scripts employed for computational analyses—including prognostic risk model construction, ROC curve generation, immune infiltration heatmaps, and pharmacogenomic analyses—as well as the raw data from immunohistochemistry (IHC) analyses, including MOD values for each individual sample, these materials have been organized and uploaded to the Figshare open-access repository. The corresponding link and DOI have been included in the revised manuscript’s Data Availability section to ensure full reproducibility.

We highly value transparency and reproducibility in research and appreciate the reviewer’s constructive suggestions. We believe these actions fully comply with PLOS ONE’s data availability requirements.https://figshare.com/articles/dataset/data_docx/29588924

*4. Recommendation of TRIPOD Guideline
(1) For a prognostic model, compliance with guidelines such as TRIPOD (Transparent Reporting of a Multivariable Prediction Model for Individual Prognosis or Diagnosis) is highly recommended and would strengthen the quality of reporting. The authors should consider mentioning or applying such guidelines when reporting their results.
Collins, G.S., Reitsma, J.B., Altman, D.G. et al. Transparent reporting of a multivariable prediction model for individual prognosis or diagnosis (TRIPOD): the TRIPOD Statement. BMC Med 13, 1 (2015). https://doi.org/10.1186/s12916-014-0241-zIF: 8.3 Q1 IF: 8.3 Q1 IF: 8.3 Q1 IF: 8.3 Q1*

***Reply:***

We sincerely thank the reviewer for this valuable suggestion. We fully agree that the TRIPOD (Transparent Reporting of a Multivariable Prediction Model for Individual Prognosis or Diagnosis) guideline is crucial for improving the clarity, transparency, and reproducibility of prognostic model studies.

In response to the reviewer’s comment, we have revised the Methods section to indicate that our reporting process is aligned with the TRIPOD recommendations [Collins et al., BMC Med, 2015]. In addition, we have added a sentence in the Discussion section to acknowledge the importance of such reporting standards and our intention to further enhance compliance in future work.

**☆ Section 2.4 (Page 5&6):**

To enhance the transparency, reproducibility, and interpretability of the prognostic model, this study adhered to the TRIPOD (Transparent Reporting of a Multivariable Prediction Model for Individual Prognosis or Diagnosis) statement [Collins et al., 2015]. The TRIPOD guideline is specifically designed to standardize the reporting of prediction model development and validation, and has been widely endorsed in biomedical research. In accordance with TRIPOD recommendations, we explicitly reported the data sources (GEO, TCGA), inclusion criteria, definition of outcomes (treatment response and survival status), variable selection process (univariate Cox regression followed by Lasso Cox regression), model construction steps, and performance evaluation metrics (Kaplan-Meier curves, AUC, ROC analysis, and calibration curves). Additionally, clinical utility was assessed using risk stratification and subgroup comparisons. While this study is retrospective in nature and lacks external validation, we have presented complete model details and ensured transparency in all analytical procedures to facilitate reproducibility.

[55] G.S. Collins, J.B. Reitsma, D.G. Altman, and K.G.M. Moons, Transparent reporting of a multivariable prediction model for individual prognosis or diagnosis (TRIPOD): the TRIPOD Statement. BMC Med 13 (2015) 1. DOI: [10.1186/s12916-014-0241-z](https://doi.org/10.1186/s12916-014-0241-z)

*(2) Figure 5 (IC50 of chemotherapeutic agents and Spearman correlation analysis):
The authors might be using a metric called “posterior Pearson” or “r JZS beta,” which is not the standard Pearson coefficient but another statistic that can have values exceeding 1. If this is the case, it needs to be clearly stated for scientific clarity: if it’s a non-standard metric, it must be thoroughly defined, justified, and its value range clearly explained in the Methods section.*

***Reply:***

Thank you for your insightful comment regarding the correlation metric presented in Figure 5. We confirm that in this figure, the correlation coefficients were calculated using **standard Spearman's rank correlation**, which ranges from –1 to 1 and measures the monotonic relationship between gene expression levels and immune cell abundance. No non-standard metrics such as "posterior Pearson" or "r JZS beta" were used. To clarify this for readers, we have revised the Methods section and added a sentence explicitly stating the correlation method and its statistical properties.

**☆ Section 2.5 (Page 7):**

Correlation analysis between risk model genes and immune cell proportions was performed using Spearman's rank correlation coefficient, as implemented in the R corrplot package. This non-parametric measure captures monotonic relationships and is widely used in immune infiltration studies. The Spearman coefficient ranges from –1 (perfect inverse relationship) to +1 (perfect direct relationship), and values exceeding this range were not observed. No non-standard correlation statistics, such as posterior Pearson or Bayesian r JZS beta, were used in this analysis.

*(3) Overall, the study is indeed an original research piece, as required by PLOS ONE. The integration of scRNA-seq, bulk RNA-seq, and IHC validation for a prognostic model in cervical cancer is a valuable approach.*

***Reply:***

We sincerely appreciate the reviewer’s recognition of the originality and methodological strength of our study. The integration of single-cell RNA sequencing (scRNA-seq), bulk RNA-seq, and immunohistochemical (IHC) validation was designed to provide a comprehensive and multi-layered perspective on radiotherapy resistance and prognosis in cervical cancer. This multi-omics approach allowed us to explore gene expression at both the cellular and tissue levels, enhancing the robustness and translational relevance of our prognostic model. We are grateful for your positive evaluation, which motivates us to continue refining our methodology and expanding its clinical applicability in future research.
